# ZNF320 is a hypomethylated prognostic biomarker involved in immune infiltration of hepatocellular carcinoma and associated with cell cycle

**DOI:** 10.18632/aging.204350

**Published:** 2022-10-26

**Authors:** Jing Zhen, Yun Ke, Jingying Pan, Minqin Zhou, Hong Zeng, Gelin Song, Zichuan Yu, Bidong Fu, Yue Liu, Da Huang, Honghu Wu

**Affiliations:** 1 Second Affiliated Hospital of Nanchang University, Nanchang, China; 2 Second College of Clinical Medicine, Nanchang University, Nanchang, China; 3 Department of Thyroid Surgery, Second Affiliated Hospital of Nanchang University, Nanchang, China; 4 Department of Science and Technology, Second Affiliated Hospital of Nanchang University, Nanchang, China

**Keywords:** ZNF320, liver cancer, biomarker, cell cycle, immune infiltrates

## Abstract

Hepatocellular carcinoma (HCC) is one of the most deadly and common malignant cancers around the world, and the prognosis of HCC patients is not optimistic. ZNF320 belongs to Krüppel like zinc finger gene family. However, no studies have focused on the influence of ZNF320 in HCC. We first analyzed ZNF320 expression in HCC by using data from TCGA and ICGC, then conducted a joint analysis with TIMER and UALCAN, and validated by immunohistochemistry in clinical HCC samples. Then we applied UALCAN to explore the correlation between ZNF320 expression and clinicopathological characteristics. Consequently, using Kaplan-Meier Plotter analysis and the Cox regression, we can predict the prognostic value of ZNF320 for HCC patients. Next, the analysis by GO, KEGG, and GSEA revealed that ZNF320 was significantly correlated to cell cycle and immunity. Finally, TIMER and GEPIA analysis verified that ZNF320 expression is closely related to tumor infiltrating immune cells (TIIC), including B cells, CD8+ T cells, CD4+ T cells, macrophages, neutrophils, and dendritic cells. The analysis of the TCGA and ICGC data sets revealed that ZNF320 expression was significantly correlated with m6A related genes (RBMX, YTHDF1, and METTL3). In conclusion, ZNF320 may be a prognostic biomarker related to immunity as a candidate for liver cancer.

## INTRODUCTION

There is evidence that hepatocellular carcinoma is a tumor with a poor prognosis and the third primary cause of cancer death in the world. Globally, the number of deaths due to HCC is estimated to be 810,000 per year, while there exit about 854,000 new cases of HCC per year [1]. Among the twenty-two most frequent cancer types, hepatocellular carcinoma (HCC) ranks sixth from the frequency perspective and fourth from the death rate associated with cancer perspective [1]. It is extremely hard to detect HCC at an early stage [2, 3]. The most common treatment for HCC is surgery, which is able to improve the survival rate, but HCC patients will still be detected with terminal cancer and have a poor prognosis. Recent existing treatments of HCC (e.g., surgery, chemoradiotherapy, hepatic transplantation, and radiofrequency ablation) can only cure a few patients, and untreated HCC patients’ mediant of overall survival (OS) is less than nine months [4]. Therefore, there is an urgent need to explore new early diagnostic markers and therapeutic targets so that the prognosis of HCC patients can be improved.

As the largest transcription factor family in the human genome, the ZNF family is widely involved in various biological processes in the human body, and zinc finger (ZNF) transcription factors are characterized by finger-like DNA domains, which require one or more zinc ions to stabilize the structure. Previous research has established the possibility that ZNF is a dominant factor in the occurrence and development of HCC [5] that ultimately regulates cell proliferation, apoptosis, invasion and metastasis by regulating the transcription of downstream target genes through a variety of regulatory levels [5, 6]. It could be a new tumor biomarker and therapeutic target for HCC [7, 8]. Though previous research has established the diagnostic and prognostic biomarkers for HCC alpha-fetoprotein (AFP), the reliability and accuracy of other biomarkers, including AFP-L3, osteopontin, and glypican-3 still need to be improved to detect HCC at an early stage [9, 10]. Zinc finger proteins are one of the most plentiful proteins in the eukaryotic system, which own exceptionally abundant biological functions, including DNA recognition, RNA transcriptional activation, apoptosis and protein structure [11]. There exists a study which objections are to confirm ZNF320 was implicated in glioblastoma [12]. However, the role and mechanism of ZNF320 in HCC have not been revealed, and its correlation with prognosis remains uncertain.

The research data in this thesis is drawn from four main sources: TCGA, GTEx, UALCAN databases, and patients and tumor specimens from 35 patients undergoing nephrectomy in the Second Affiliated Hospital of Nanchang University from June 2017 to January 2021. In this study, we dissected the expression of ZNF320 mRNA and protein in HCC and assessed the correlation between the expression level of ZNF320 and prognosis of HCC. Besides, we also investigated the possible mechanism of high expression of ZNF320 in HCC and the relation of ZNF320 expression and cell cycle, tumor infiltrating immune cells [3, 13] in HCC patients. The importance and originality of this study are that it reveals the important function of ZNF320 in hepatocellular carcinoma and provides a potential link between ZNF320 and cell cycle, HCC immune invasion, m6A Modification and its underlying mechanisms.

## MATERIALS AND METHODS

### Data collection and processing

Hepatocellular carcinoma (HCC) clinical and mRNA expression level was collected from the TCGA Database. In terms of the gene expression profile, this study included 374 LIHC samples and 50 normal samples, and the data type of mRNA expression profile was HTSeq-FPKM. We obtained clinical information from 377 patients. In addition, RNA-seq data and clinical information were also acquired from the ICGC website (https://dcc.icgc.org/projects/LIRI-JP). [LINC-JP] Liver Cancer - NCC, JP datasets included 202 normal samples and 243 tumor samples [14].

### Patients and tumor specimens

Human HCC tissues and corresponding adjacent tissues were obtained from 35 patients undergoing nephrectomy in the Second Affiliated Hospital of Nanchang University from January 2018 to January 2021. The patients’ informed consent was obtained. At the same time, the research ethics committee of Second Affiliated Hospital of Nanchang University agreed to the experiment.

### TIMER database analysis

The Tumor Immune Estimation Resource (TIMER) is a synthetic web used for analyzing the levels of immune invasion in diverse cancers, which includes 32 cancer types. In this work, we use “Diff-Exp module” to confirm the expression of ZNF320 in diverse tumors. Then the “gene module” was used to estimate the relationship of ZNF320 with immune infiltration. Moreover, the “SCNA module” was used to compare the level of tumor invasion among tumors with different somatic copy number changes for ZNF320. Finally, we considered the correlation between ZNF320 expression in TIICs and immune markers by using related modules. The difference between infiltration level and normal level was evaluated for each SCNA category through Wilcoxon rank-sum test. Finally, with the help of the “Correlation module”, considering the Spearman’s rho value and predicted statistical implications, we verified the relationship between ZNF320 and tumor infiltrating immune cell markers in HCC [15].

### Immunohistochemistry

Cut formalin-fixed, paraffin-embedded HCC tissue into 4 μm-thick sections. After deparaffinization, rehydration, and microwave heating in microwave-heated antigen unmasking solution (EDTA, pH8.0) to repair the antigen, 30 minutes in total was used to seal the sections with goat serum. Next, incubate the sections overnight with anti-ZNF320 monoclonal antibody (RRID 24882-1-AP, Abcam, 1:500 dilution) at 4° C. After that, HRP-conjugated secondary antibody (Boster) was allowed to stand at room temperature for 2 hours. Subsequently, the two-step method (catalog no.: PV-9000; ZSGB-BIO Co., Ltd., Beijing, China) were used for immunostaining. Finally, three pathologists who are unaware of the clinical parameters assessed the staining intensity and the percentage of positive cells semi-quantitatively.

### UALCAN database analysis

UALCAN database is a tumor data on-line analysis website that provides comprehensive cancer transcriptome and clinical patient data (extracted from TCGA) [16]. In our research, we assessed ZNF320 expressions by using the “Expression Analysis” module. We also studied the correlation between the ZNF320 expression and clinicopathological features.

### LinkedOmics analysis

LinkedOmics, a comprehensive online site, is usually chosen to analysis multidimensional data within and across 32 kinds of cancer. Using it, we succeeded in mining the co-expressed genes linked to ZNF320 in the TCGA LIHC database through the results of analysis. Volcano plots and heat maps provided strong evidence for this. We define the top 500 related genes as co-expressed genes.

### DAVID

Database for Annotation, Visualization and Integrated Discovery (DAVID) 6.8 is a comprehensive, functional annotation website, which enables us to study the biological functions and signal pathways of specific gene sets. Gene annotation includes Gene Oncology (GO) and Kyoto Encyclopedia of Genes and Genomes (KEGG) pathway analysis [17].

### DNA methylation

The cBioPortal database provides visualization, analysis, and download of large-scale tumor genome data for a variety of cancers. We used it to study the association between ZNF320 promoter methylation level and expression in HCC with “Plot module” [18].

### Gene set enrichment analysis (GSEA)

We used GSEA to conduct a group study on the RNA-sequencing data of 374 TCGA-LIHC patients downloaded from Genomic Data Commons, analyzing the pathways associated with ZNF320 expression in HCC specimen. The number of permutations: 1000; normalized enrichment score (NES) >1; false discovery rate (FDR) <0.25, p-val<0.05 [19].

### PPI network construction

STRING database is an online tool for evaluating protein interactions in multiple ways. In our research, the top 500 genes with co-expression coefficients greater than 0.4 were collected from the STRING database. They were evaluated by Cytoscape 3.8.2 and its plug-in MCODE (Molecular Complex Detection). And the selection criteria are as follows: Max depth=100, node score cutoff=0.2, K-core=2 [20].

### Validation of hub genes

Hub genes are highly connected to the nodes in the module and have been certificated to play an important role in function. The significance of the genes was measured by the absolute values of the Spearman’s correlation in our study. On the basis of the results, the top 500 genes with confidence > 0.9 were uploaded to the STRING database to construct protein-protein interaction (PPI). Then, we used Cytoscape 3.8.0 and its plug-in MCODE to evaluate the top 500 genes. Furthermore, a standard for hub genes (yellow nodes) was set with a degree cut-off = 2, node score cut-off = 0.2, k-core = 2, and max. depth= 100, which was screened with MCODE. In total, there were 6 genes scoring the highest and initially defined as hub genes. Ultimately, after digging out their backgrounds, three genes, closely related to the cell cycle, were considered to be a “real” hub gene among these genes.

### GEPIA analysis

GEPIA [21], an interactive web server containing RNA sequencing data based on the TCGA and GTEx databases, which analyzed the mRNA expression. In this study, GEPIA was used to compare tumors with normal tissues, analyze pathological stages, and analyze m6A related prognosis. We used Spearman method to ascertain the correlation coefficient of relevance [22].

### Statistical analysis

All statistical analysis in our work was done by R software (version 3.6.3). The detection of different ZNF320 expression levels between LIHC samples and normal samples was realized by using the “limma” and “beeswarm” packages of “R” and rank sum test method. We probed the relevance between ZNF320 expression and clinicopathological feature by Wilcoxon signed-rank test or Kruskal-Wallis test. Then, Kaplan-Meier survival curve using log-rank test to explore the survival distribution among patients with different levels of expression was drawn to evaluate whether the differential genes expression had a significant influence on the prognosis of patients (p<0.05). Univariate and Multivariate Cox regression analysis screened factors significantly related to prognosis (p<0.05) (Cox model uses the “survival” and “survminer” packages of “R”). Lastly, we used the ROC curve drawn by “survival ROC” to evaluate the predictive ability of ZNF320 expression level on one-year, three-year, or five-year survival. T-test was used to study the different expression of m6A interrelated genes between ZNF320 high and low expression groups.

### Data availability

The expression profiling of genes from diabetics and diabetics nephropathy can be available from the GEO (GSE142153 and GSE26168). The other data can be obtained from the corresponding author (Bo Zhang).

## RESULTS

### ZNF320 is over expressed in HCC

**Figure 1:**
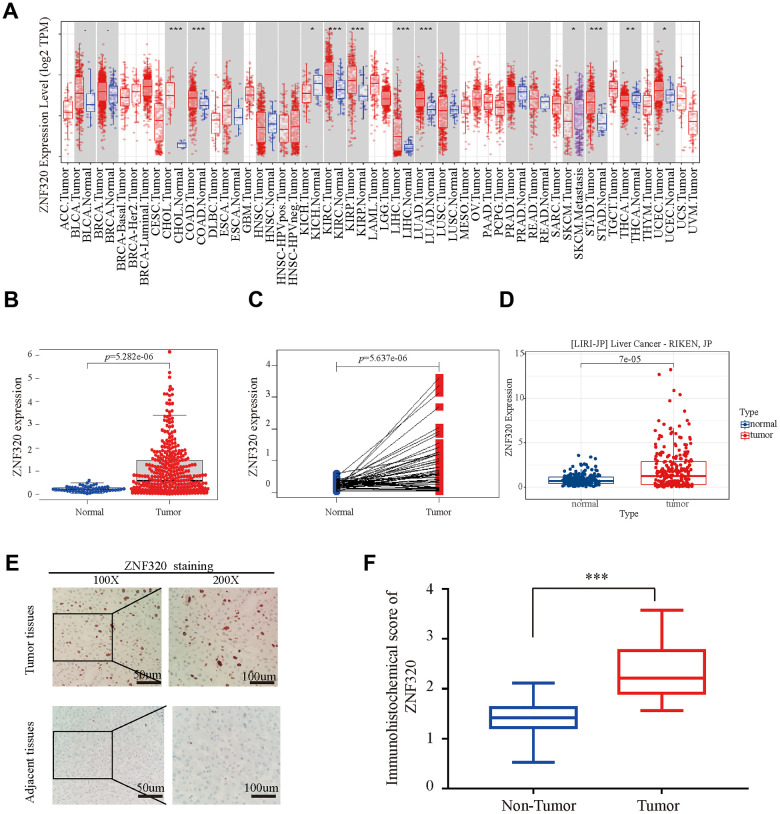
**ZNF320 is over expressed in HCC tissues.** (**A**) The mRNA level of ZNF320 in 33 kinds of tumor types by TIMER. (*p<0.05,**p<0.01,***p<0.001). (**B**) Expression levels of ZNF320 were higher than the corresponding normal tissues in HCC samples (p=5.282e-06). (**C**) ZNF320 expression in HCC group downloaded from TCGA RNA-seq dataset (p=5.637e-06). (**D**) The mRNA expression level of ZNF320 in tumor and normal tissues in the UALCAN (**E**, **F**) Typical images of immunohistochemistry (IHC) in 35 pairs of HCC tissues showing the protein expression of ZNF320 in HCC and adjacent nontumor tissues.

To compare the differential expression of ZNF320, we first studied the expression of ZNF320 in LIHC and other tumors by using TIMER database. Expression of ZNF320 in liver hepatocellular carcinoma was predicted to be vitally upregulated (Figure 1A). Next, we used TCGA sequencing data to explore ZNF320 expression in 374 HCC samples and 50 normal samples. Our result revealed ZNF320 expression was vitally upregulated in HCC (Figure 1B). Analysis of the paired samples revealed a notable rise of ZNF320 expression in HCC tissues (n =50) (Figure 1C). What’s more, we used ICGC, another database, to do the same analysis (Figure 1D), and got a similar consequence with our preliminary result in TCGA. To further check the ZNF320 expression in HCC, we compared 35 HCC tissue samples with para cancerous samples by IHC analysis. Our results showed that ZNF320 expression protein in HCC tissues was conspicuously higher than that of adjacent tumor tissues (Figure 1E, 1F). In sum, these outcomes verified that ZNF320 is conspicuously overexpressed in HCC tissues.

### Association of ZNF320 expression and clinicopathological characteristics in HCC patients

**Figure 2:**
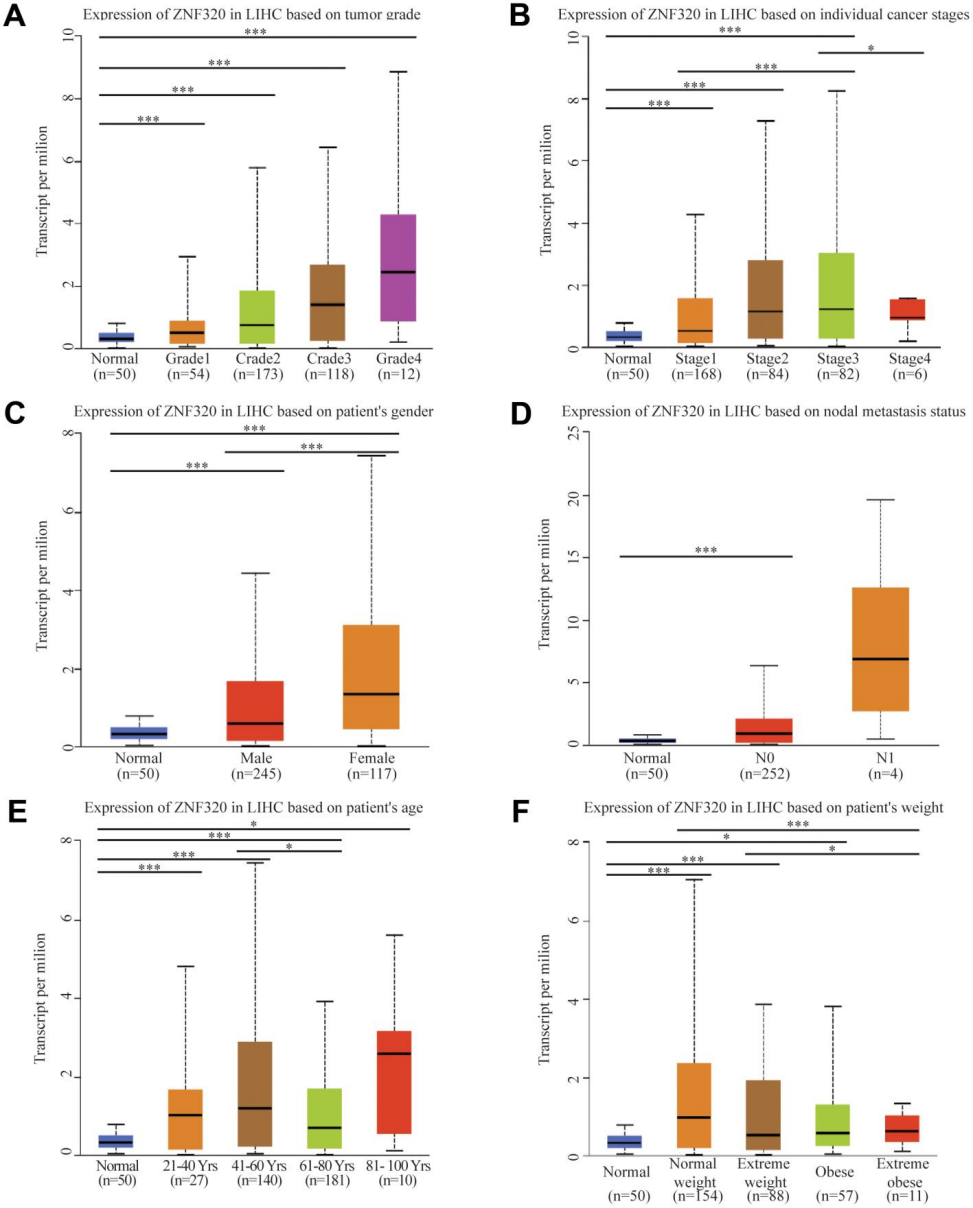
**ZNF320 expression is a correlation with clinicopathological characteristics.** Increased ZNF320 expression was significantly with (**A**) higher grade, (**B**) stage, (**C**) nodal metastasis, (**D**) gender, (**E**) age, and (**F**) weight.

After the expression of mRNA and protein were found to increase in HCC patients, we next used UALCAN to integrate various clinic factors of HCC samples, including patients’ individual tumor grade, cancer stages, gender nodal metastasis, age, and weight. Then we compare the expression levels of ZNF320 in each group. The result exhibited that with grade increasing, the patients had statistically higher expression of ZNF320. (Figure 2A) Additionally, ZNF320 were associated with patients’ cancer stages, and patients in more advanced cancer stages has a tendency to have higher expression of ZNF320. (Figure 2B) We also caught that the expression of ZNF330 increased in tumor tissue with nodal metastasis. (Figure 2C) The analysis result revealed significant differences between male and female. (Figure 2D) What’s more, compared with the normal subjects, older and heavier patients maintained a high expression level of ZNF320. (Figure 2E, 2F) The above findings further prove that ZNF320 is increased expression in HCC and related to clinicopathological characteristics.

### ZNF320 expression is an independent prognostic factor which is associated with poorer prognosis of HCC patients

**Figure 3:**
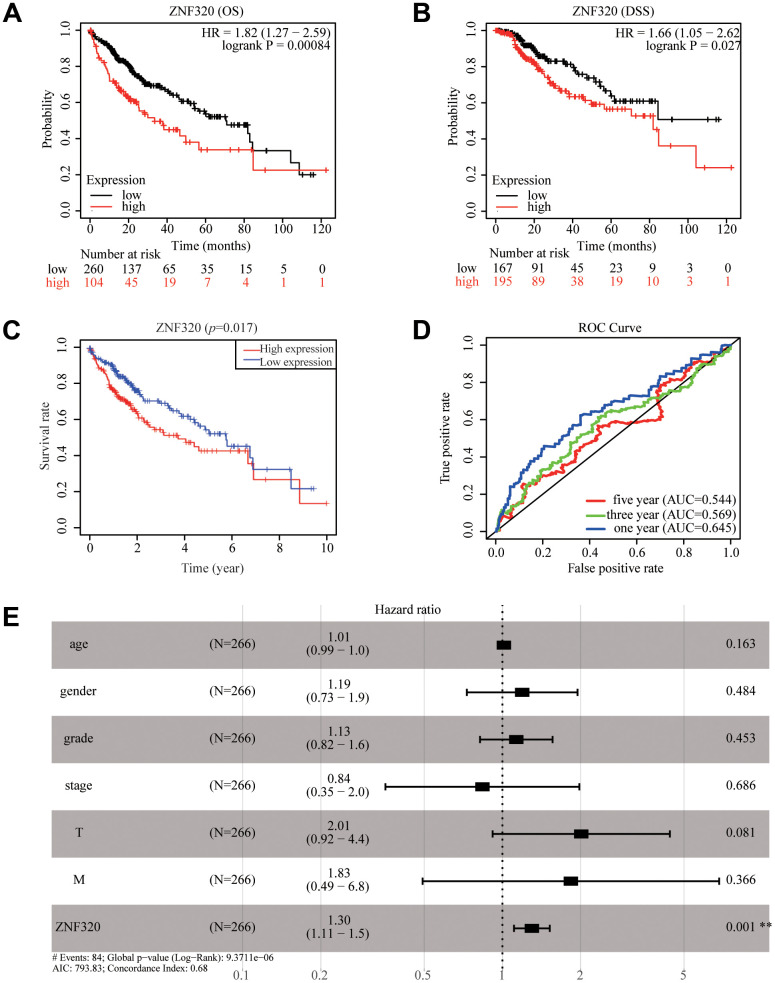
**ZNF320 expression in tumor tissues is associated with poor survival in HCC patients.** (**A**, **B**) HCC patients with lower expression level of ZNF320 had favorable OS and DSS results from KM plotter (p=0.00084; p=0.027). (**C**) HCC patients with lower expression level of ZNF320 had favorable OS results from R(p=0.017) (**D**) ROC curves for the 1-, 3-, and 5-year survival according to the expression level of ZNF320. AUC, area under the curve; ROC, receiver operating characteristic. (**E**) A forest plot of the results of the multivariate analysis. *, P<0.05; **, P<0.01; ***, P<0.001. HR, hazard ratio; CI, confidence interval; T, tumor; N, node, M, metastasis; OS, overall survival; AIC, Akaike’s information criterion.

To further find the potential role of ZNF320 expression in TCGA patients with Hepatocellular Carcinoma, we used Kaplan Meier survival method for survival analysis. We found via the KM plotter that high expression of ZNF320 patients had shorter OS and DSS times than low expression (OS:HR= 1.82, P<0.01; DSS:HR=1.66, P<0.05) (Figure 3A, 3B). The results also manifested that patients with high ZNF320 expression had a obviously shorter OS time proportionate to low expression group, which meant that ZNF320 high expression predicted a poorer prognosis (p = 0.017) (Figure 3C). Next, we constructed the ROC curve to detection the sensitivity and specificity to predict one-, three-, and five- year survival in HCC patients, The AUC of the ROC curve is significant, (one-year AUC:0.645, three-year AUC:0.569, five-year AUC:0.544), which indicates that the expression of ZNF320 can availably predict the survival time of patients (Figure 3D). Then, to screen out deeply the connections between the ZNF320 expression and clinical characteristics, we conducted univariate and multivariate Cox regression (Table 1). The univariate cox analysis revealed ZNF320 (HR: 1.298, 95% CI: 1.127-1.495, p <0.001) is substantially related with OS. The multivariate cox analysis exposed the variables of ZNF320 expression (HR: 1.294, 95% CI: 01.108-1.521, p = 0.001) could regard as an independent prognostic indicator for patients with HCC. The forest map also demonstrated this point (Figure 3E). In brief, our study implied that ZNF320 expression can be an independent prognostic parameter, and cases with elevated ZNF320 expression tend to be communicate with a worse prognosis.

**Table 1:** Univariate and multivariate COX regression analysis of factors associated with OS in Liver cancer patients.

**Variable**	**Univariate analysis**				**Multivariate analysis**		
	**HR**	**95%CI**	**P-value**		**HR**	**95%CI**	**P-value**
age	1.007	0.990-1.024	0.441		1.013	0.995-1.031	0.163
gender	0.839	0.536-1.314	0.443		1.192	0.729-1.947	0.484
grade	1.073	0.795-1.449	0.645		1.131	0.820-1.560	0.453
stage	1.809	1.426-2.294	<0.001		0.837	0.354-1.978	0.686
T	1.767	1.415-2.207	<0.001		2.011	0.917-4.414	0.081
M	3.892	1.223-12.386	0.021		1.834	0.492-6.829	0.366
ZNF320	1.298	1.127-1.495	<0.001		1.298	1.108-1.521	0.001

OS, overall survival; HR, hazard ratio; CI, confidence interval; T, tumor; N, node; M, metastasis; Bold values indicate P-values<0.05.

### Correlation between ZNF320 mutation, hypermethylation, and prognosis in HCC

**Figure 4:**
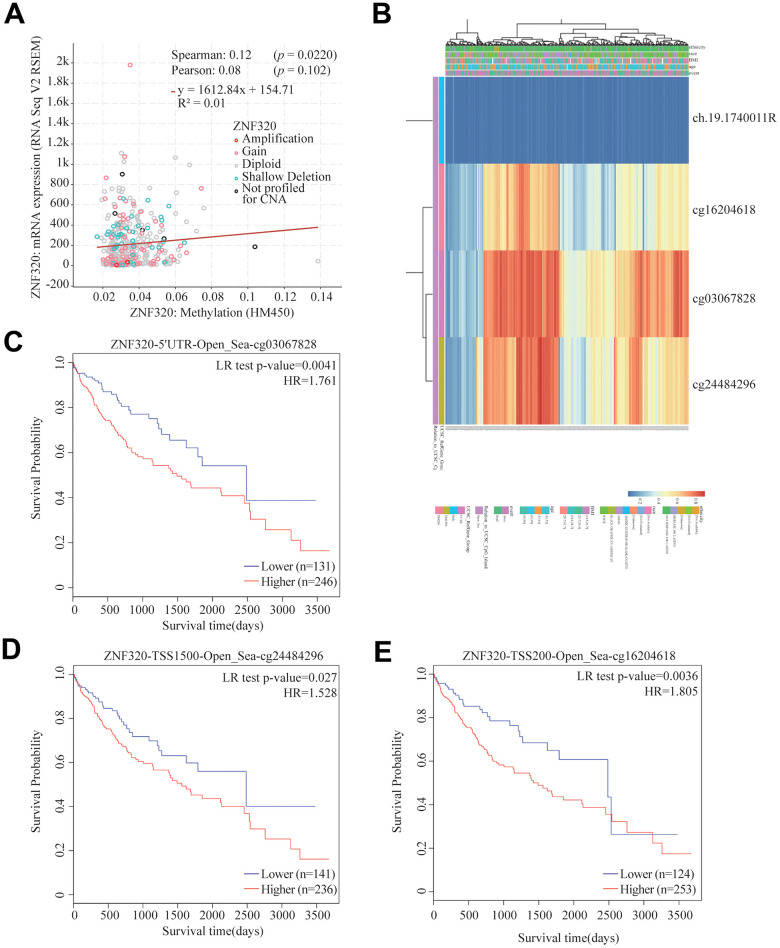
**Correlation between ZNF320 mutation, hypermethylation, and prognosis in HCC.** (**A**) The correlation between ZNF320 methylation and its expression level. (**B**) The visualization between the methylation level and the ZNF320 expression. (**C**–**E**) the Kaplan–Meier survival of the promoter methylation of ZNF320.

Significant increases of ZNF320 expression in HCC were observed. In consequence, we will study the reason for the overexpression of ZNF320. As methylation plays an important role in gene expression. we studied the methylation and expression of ZNF320 through the cBioPortal dataset. The finding was that ZNF320 expression was highly positively correlative with methylation (R = 0.1, p =0.022) in HCC (Figure 4A). We detected the methylation level of FARSB promoter in liver cancer tissues through the MethSurv website, and the correlation heat map showed that three methylation probes 16204618, cg03067828, and cg24484296 were in a hypermethylated state (Figure 4B) What’s more, the MethSurv analysis indicated that patients with high ZNF320 methylation had a worse overall survival than those with low ZNF320 methylation (p < 0.05) (Figure 4C–4E).

In conclusion, the methylation level of ZNF320 was positively associated with its expression. It is supposed that the increased methylation level of ZNF320 promoter leads to the high ZNF320 expression in liver cancer, which in turn causes poor prognosis in patients with liver cancer.

### Function and pathway enrichment analyses and co-expression genes of ZNF320 in HCC

**Figure 5:**
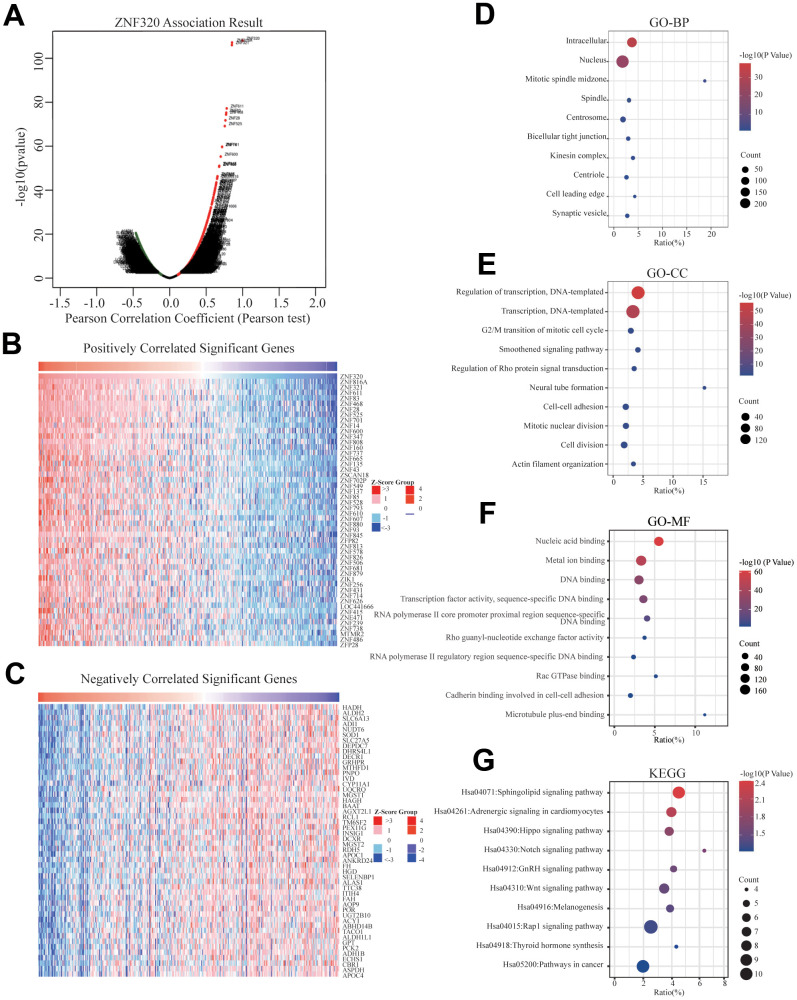
**Genes differentially expressed in correlation with ZNF320 and enriched GO annotations, KEGG pathway of ZNF320 correlated genes in HCC.** (**A**) Pearson test was used to analyze the association between ZNF320 and genes differently expressed in HCC, red indicates positively correlated genes, and green indicates negatively correlated genes. (**B**, **C**) The top 50 genes positively and negatively relative to ZNF320 in HCC were shown by heat maps. (**D**–**F**) Significant Gene Ontology terms of the top genes most positively relative to ZNF320, including biological processes (BP), molecular function (MF), and cell component (CC). (**G**) Significant KEGG pathways of the top genes most positively associated with ZNF320.

**Figure 6:**
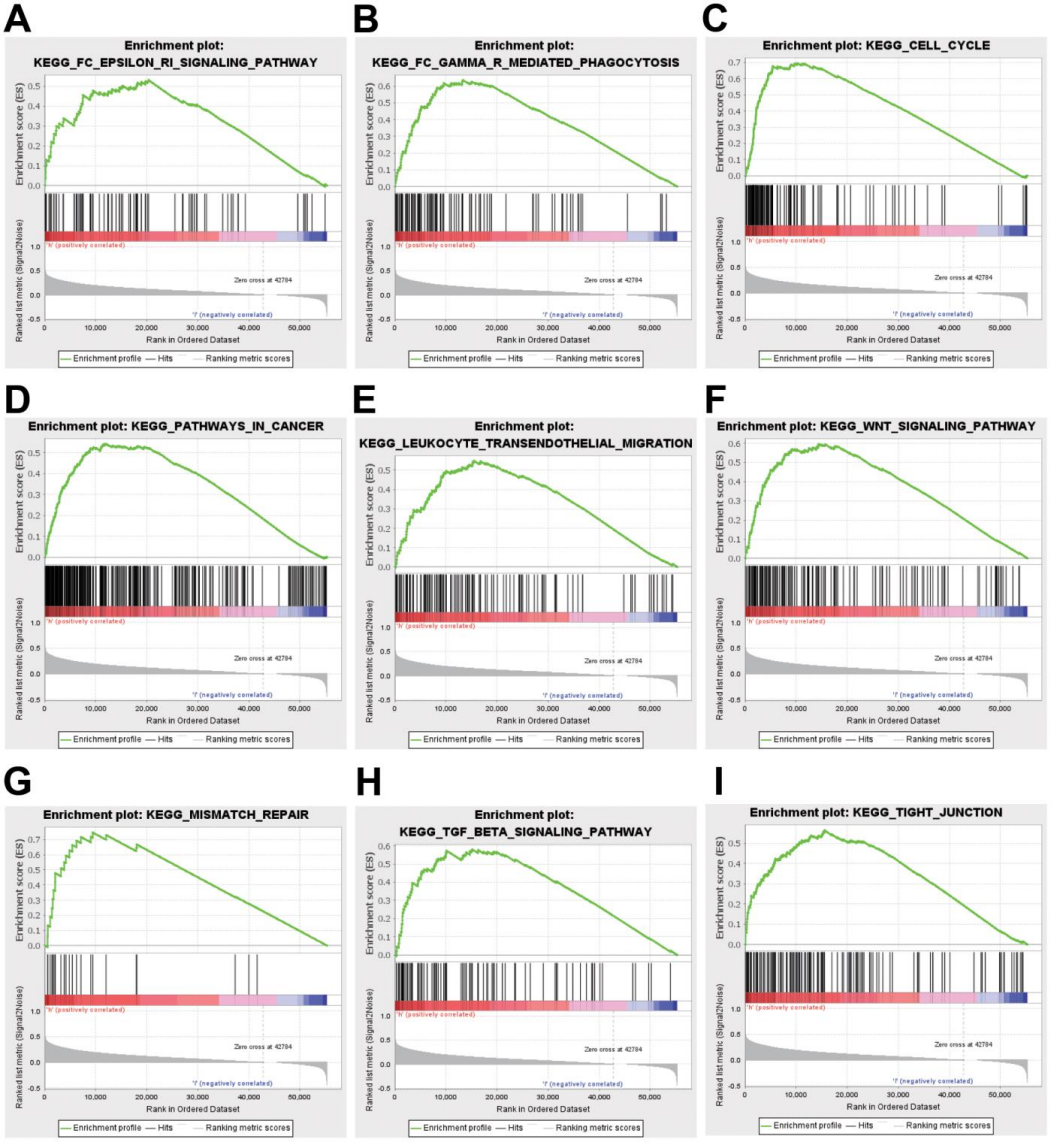
GSEA was used to validate the gene signatures, including positive regulation of (**A**) FC epsilon pathway, (**B**) FC gamma R mediated phagocytosis, (**C**) cell cycle, (**D**) pathway in cancer, and (**E**) leukocyte transendothelial migration, (**F**) WNT signaling pathway, (**G**) mismatch repair, (**H**) TGF-beta signaling pathway, (**I**) tight junction.

To better realize the biological meaning of ZNF320 in HCC, the LinkedOmics was used to find the co-expression pattern of ZNF320. As displayed in Figure 5A, it reveals that 13934 genes (red dots) positively associated with ZNF320, and 5989 genes (green dots) was negative correlation with ZNF320 (*p*-value < 0.05). The top 50 obvious gene set positive (left) and negative (right) correlation with ZNF320 were present in the heatmap. (Figure 5B, 5C) The top 200 genes that were correlated most clearly with ZNF320 were extracted for enrichment analysis. Moreover, Supplementary Table 1 detailed lists the co-expressed genes. We used David database to analyze the function and pathway enrichment of gene co-expressed with ZNF320 (version 6.8). Bubble plots demonstrate the top 10 enriched biological functions and pathways (based on *P*-value) were present by the *hipplot.* Analysis using the GO database showed that ZNF320 was functionally associated with G2/M transition of mitotic cells, Cell-cell adhesion, Regulation of transcription, Cell division, Cadherin binding involved in cell-cell adhesion, DNA binding, Transcription factor activity (Figure 5D–5F). Pathway analyses were conducted by the KEGG database. Results revealed that genes were enriched mostly in pathways in cancer, Wnt signaling pathway. These pathways, which were related to the tumorigenesis and progression of tumors, indicated that ZNF320 may be closely-related to HCC tumorigenesis and progression. (Figure 5G) Furthermore, GSEA was conducted to seek out KEGG pathways, which exposed FC epsilon pathway, FC gamma R mediated phagocytosis, cell cycle, WNT signaling pathway, and pathways in cancer, leukocyte transendothelial migration, mismatch repair, tight junction (Figure 6A–6I) These results suggested that ZNF320 worked by participating in cell cycle, DNA mismatch repair, WNT signaling pathway and immune-related pathways in HCC.

### Correlation of ZNF320 expression with cell cycle

**Figure 7:**
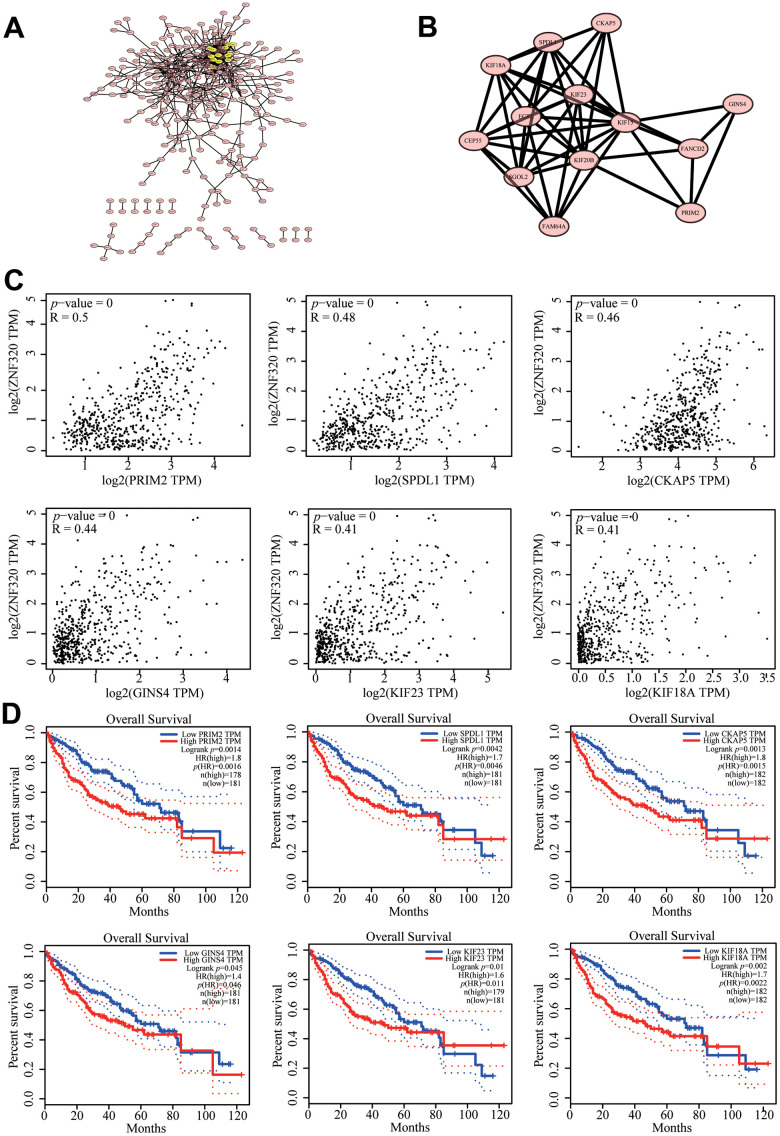
**Protein–protein interaction network of related genes (Top200) and analysis of hub genes in HCC.** (**A**) Protein–protein interaction (PPI) network (**B**) MCODE analysis (**C**) Correlation between ZNF320 and the mRNA expression of SPDL1, KIF18A, PRIM2, GINS4, KIF23, CKAP5 in HCC determined using GEPIA (**D**) Prognosis analysis of correlational genes.

To further investigate the potential function of ZNF320 in HCC, STRING database was performed on ZNF320 co-expressed genes to make the protein–protein interaction (PPI) network, and Cytoscape (MCODE plug-in) was applied to find the most vital module. What’s more these genes were highlighted in yellow (Figure 7A). Based on the degree score, the module with the highest score consisted PRIM2, SPDL1, CKAP5, GINS4, KIF23, KIF18A. (Figure 7B) And we investigate that there existed an obvious correlation coefficient between ZNF320 and PRIM2 through GEPIA analysis (Figure 7C). What’s more, we have done prognosis analysis of these genes by Kaplan-Meier Survival Method, which showed that all of these 6 genes were oncogenes that were related to poor prognosis. (Figure 7D) The results of pathway analysis proved that all 6 genes in the module were related to cell cycle, and based on the above analysis, we inferred that the impact of ZNF320 on HCC prognosis may be connected to the cell cycle.

### ZNF320 correlates with tumor purity and immune infiltration level in HCC

**Figure 8:**
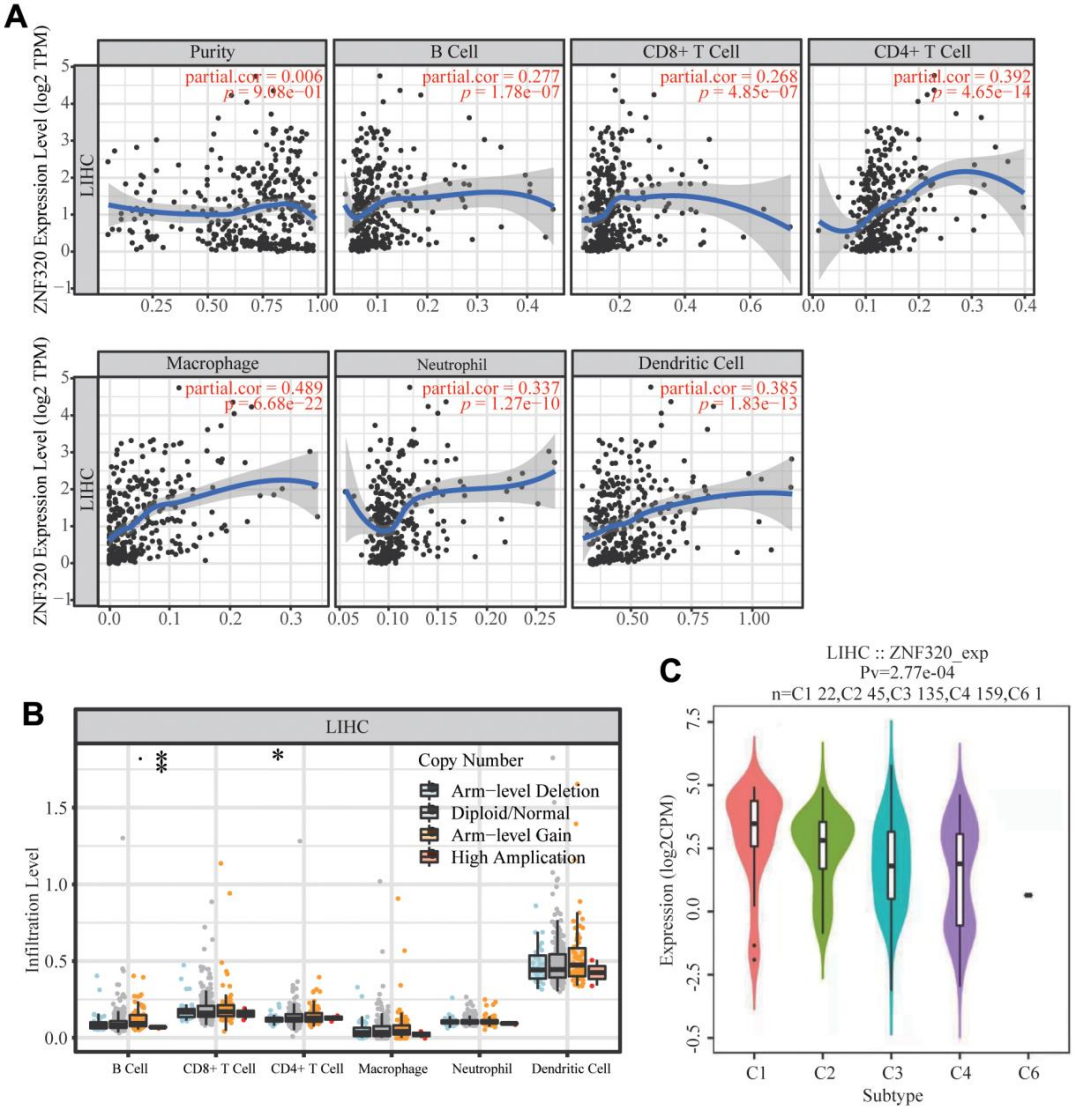
**Correlations of ZNF320 expression with immune infiltration level in HCC (TIMER).** (**A**) ZNF320 expression is positively related to tumor purity, infiltrating levels of CD8 +T cells, CD4 + T cells, macrophages, neutrophils and dendritic cells in HCC. (**B**) ZNF320 CNV affects the infiltrating levels of CD8 + T cells, macrophages, and dendritic cells in HCC. *P < 0.05, ***P < 0.001. (**C**) Distribution of ZNF320 expression across immune subtypes in LIHC (TISIDB). The different color plots represent the five immune subtypes (C1: wound healing; C2: IFN-gamma dominant; C3: inflammatory; C4: lymphocyte-depleted and C6: TGF-b dominant).

It has been confirmed that the occurrence and development of tumors and their prognosis depend on the number of immune cell infiltration. Meanwhile, KEGG and GSEA suggest that ZNF320 is associated to immune infiltration. Through analyzing whether ZNF320 expression was associated to immune infiltration levels in HCC, we discovered a positive correlation between ZNF320 expression and tumor purity, infiltrating levels of B cells, CD8+ T cells, CD4 + T cells, Macrophage, Neutrophils, and Dendritic cell (Figure 8A). Furthermore, ZNF320 CNV was significantly correlate with infiltrating levels of B cells and CD4 + T cells. (Figure 8B). Additionally, we detected the expression of ZNF320 in different immune subgroups in LIHC by TISIDB. We found higher ZNF320 expression in the Cl (wound healing) and C2 (IFN-gamma dominant) subgroups, whereas lower in the C6 (TGF-b dominant) subgroup (Figure 8C). Altogether, ZNF320 is closely related with the main immunity cells in HCC.

### The correlation between ZNF320 and markers of immune infiltrates in HCC

**Figure 9:**
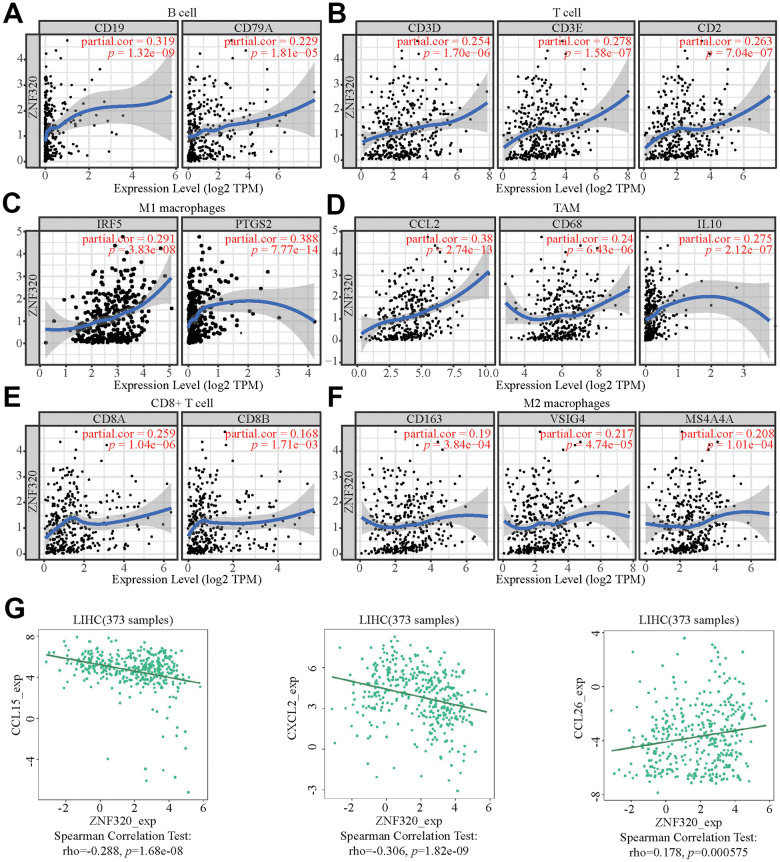
**ZNF320 expression correlates with marker sets of immune cells in HCC.** The association between the expression levels of ZNF320 and B cell (**A**), T cell (**B**), M1 macrophages (**C**), TAM (**D**), CD8+T cell (**E**), and M2 macrophages (**F**) using TIMER. (**G**) The association between the expression levels of ZNF320 and the HCC-related chemokines.

To further explore the underlying correspondence of ZNF320 and different immunocyte, TIMER was used to analyze correlations between ZNF320 levels and multiple gene markers of immune cells, which included TAM, M1 and M2 macrophages, CD8+ T cell, T cell and B cell in HCC (Table 2). Because B cells, T cells, CD8+ T cells, and macrophages are the most relevant immune cell types to ZNF320 expression, the connection between ZNF320 and the sets of immune markers of these cells were further investigated through TIMER. We found positive correlations between ZNF320 and the expression of these specific immune markers of these cells, e.g., B cell markers, TAM markers, M1 markers, M2 markers (Figure 9A–9F). In conclusion, our results showed that there is an association between ZNF320 and tumor cell infiltration in HCC.

**Table 2:** Relationship between ZNF320 and gene marker set of different immune cells using the TIMER database.

**Description**	**Gene markers**	**LIHC**			
		**Purity**		**None**	
		**Cor**	**P**	**Cor**	**P**
B cell	CD19	0.296	6.25E-09	0.298	1.55E-08
	CD79A	0.191	0.000207	0.197	2.34E-04
T cell (general)	CD3D	0.211	4.30E-05	0.225	2.55E-05
	CD3E	0.210	4.79E-05	0.226	2.22E-05
	CD2	0.202	9.08E-05	0.222	3.21E-05
CD8+ T cell	CD8A	0.208	5.76E-05	0.227	2.12E-05
	CD8B	0.133	1.03E-02	0.150	5.11E-03
Monocyte	CD86	0.332	6.73E-11	0.346	4.88E-11
	CSF1R	0.264	2.64E-07	0.286	6.91E-08
TAM	CCL2	0.314	7.28E-10	0.006	9.08E-01
	CD68	0.213	3.62E-05	0.320	1.36E-09
	IL10	0.237	3.82E-06	-0.472	1.31E-20
M1	IRF5	0.311	9.58E-10	0.291	3.65E-08
	PTGS2	0.318	3.54E-10	0.333	2.22E-10
M2	CD163	0.148	4.34E-03	0.006	9.08E-01
	VSIG4	0.173	8.41E-04	0.164	2.25E-03
	MS4A4A	0.153	3.09E-03	0.187	4.97E-04
Neutrophils	CEACAM8	0.043	4.14 E-01	0.047	3.97E-01
	ITGAM	0.258	4.91E-07	0.274	2.60E-07
	CCR7	0.157	2.35E-03	0.164	2.15E-03
Natural killer cell	KIR2DL1	0.056	2.81E-01	0.029	5.94E-01
	KIR2DL3	0.143	5.81E-03	0.151	5.02E-03
	KIR2DL4	0.102	4.94E-02	0.095	7.50E-02
	KIR3DL1	0.047	3.62E-01	0.032	5.59E-01
	KIR3DL2	0.131	1.15E-02	0.149	5.62E-03
	KIR3DL3	0.050	3.32E-01	0.042	4.34E-01
Dendritic cell	HLA-DPB1	0.258	5.38E-07	0.264	6.61E-07
	HLA-DQB1	0.161	1.94E-03	0.167	3.04E-06
	HLA-DRA	0.256	6.15E-07	0.268	1.38E-08
	HLA-DPA1	0.245	2.02E-06	0.260	3.62E-14
	CD1C	0.278	5.24E-08	0.271	4.17E-11
	NRP1	0.359	1.34E-12	0.346	1.74E-23
	ITGAX	0.288	1.86E-08	0.288	6.74E-45

Moreover, ZNF320 was also associate with the HCC-related chemokines including CCL15, CCL8, and CCL26. It could be known from the results that ZNF320 was negatively associated with CCL15 and CXCL2 while positively associated with CCL26 (Figure 9G). Therefore, ZNF320 constitutes an immunosuppressive microenvironment by affecting the expression levels of relevant chemokines, helping tumor cells to participate in immune escape, thereby enhancing tumor cell invasion and migration capabilities.

These results indicated that ZNF320 could affect immune cell infiltration partly by regulating these chemokines expression.

### Prognostic analysis of ZNF320 expression in HCC in view of immune cells

**Figure 10:**
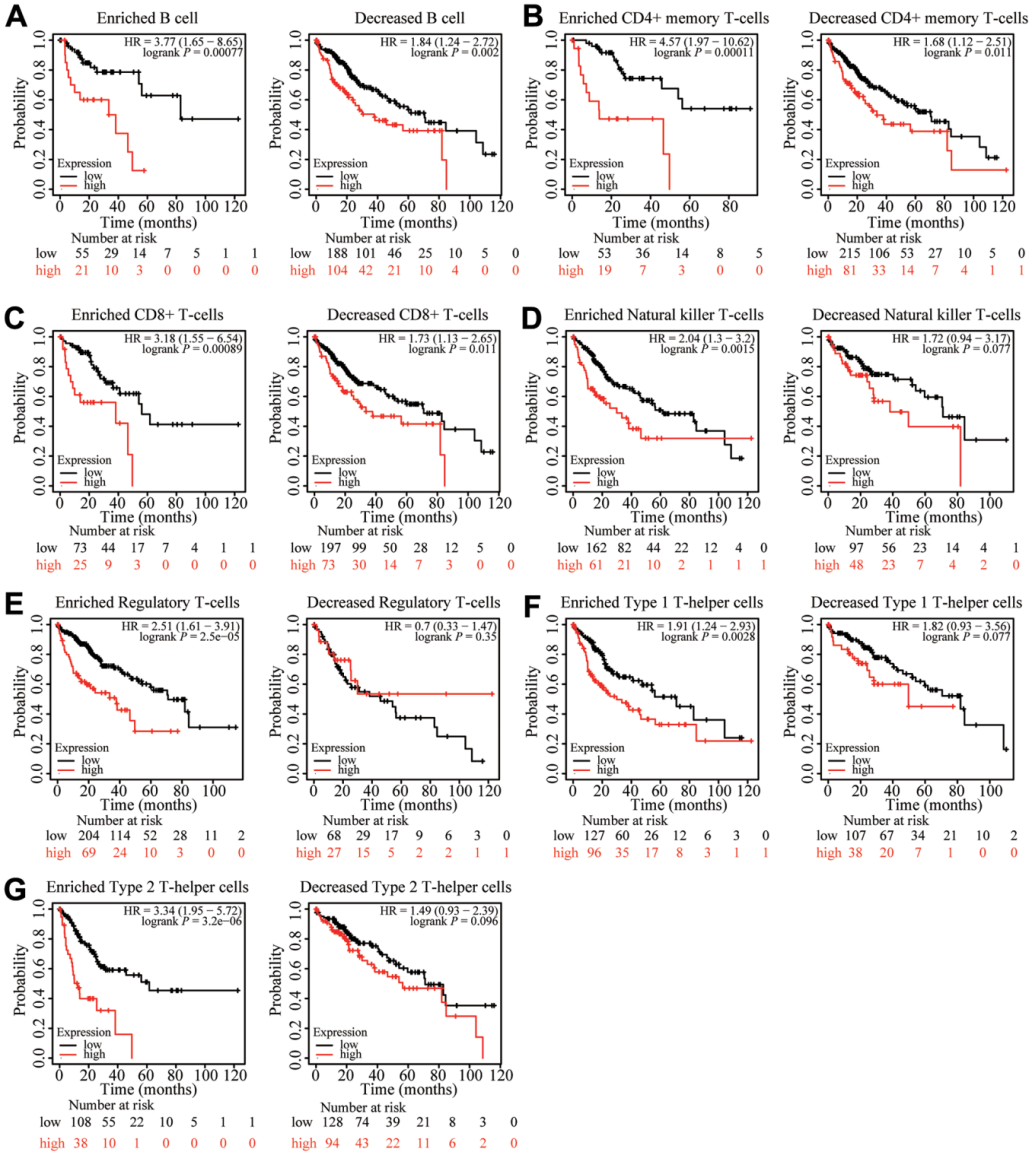
**Kaplan-Meier survival curves according to high and low expression of ZNF320 in immune cell subgroups in liver cancer.** (**A**–**G**) Correlations between ZNF320 expression and OS in different immune cell subgroups in HCC patients were estimated by Kaplan-Meier plotter.

Because ZNF320 expression was significantly correlated with immune infiltration, which is related to prognosis in HCC, we analyzed whether ZNF320 expression influences the prognosis of HCC by influencing immune infiltration. The prognosis was analyzed based on the expression level of ZNF320 in HCC related immune cell subgroup. As shown in Figure 10, the expression of ZNF320 in different B cell, CD4+ memory T-cells, CD8 + T cell levels had no significant correlation with the prognosis of HCC. Whether or not these immune cells are enriched or not high expression of ZNF320 leads to a poor prognosis for HCC patients (Figure 10A–10C). However, when Natural killer T-cells, Regulatory T-cells, Type 1 T-helper cells, and Type 2 T-helper cells are enriched, ZNF320 high expression leads to a poor prognosis in patients with HCC (Figure 10D–10G). These outcomes indicated that differential gene expression can affect patient outcomes at different levels of cell infiltration. In conclusion, these results proclaimed that ZNF320 was related to tumor cell infiltration in HCC.

### Relationship between ZNF320 Expression and m6A modification in HCC

**Figure 11:**
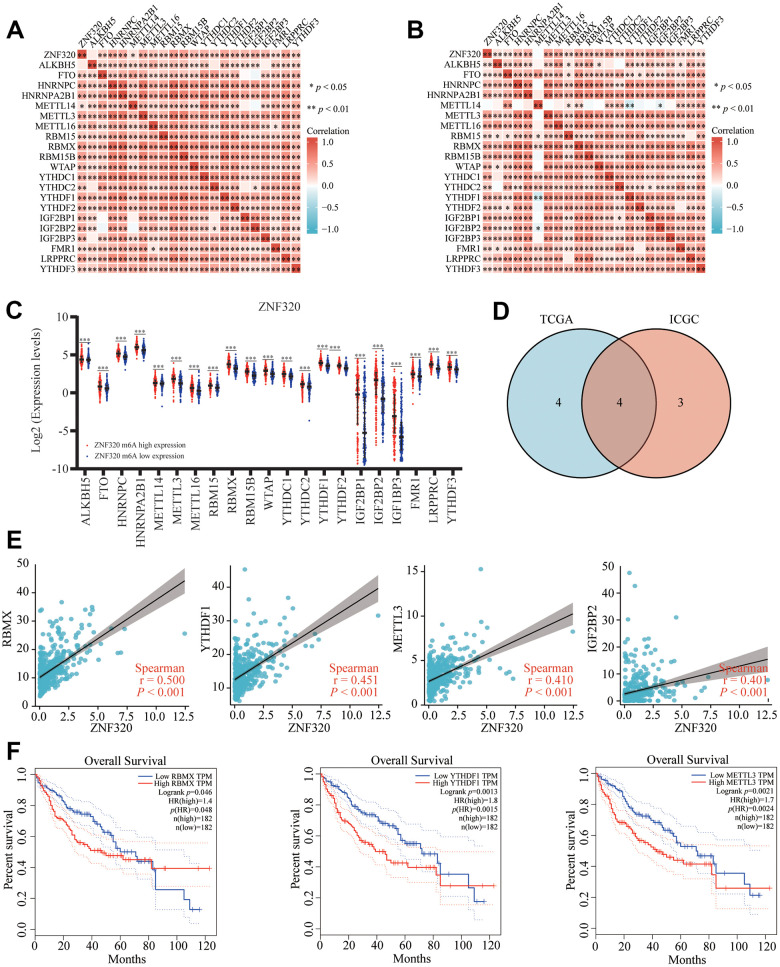
**Correlation of ZNF320 expression with m6A related genes in HCC.** (**A**, **B**) TCGA HCC data set and ICGC data set analyzed the correlation between the ZNF320 and m6A related genes expression in HCC. (**C**) The differential expression of glycolysis related genes between high and low ZNF320 expression groups in HCC tumor samples. (**D**) Venn diagram showed correlation coefficient greater than 0.39 with ZNF320 in two databases, including RBMX, YTHDF1, METTL3, IGF2BP2. (**E**) Draw a scatter plot to show the correlation between the ZNF320 and glycolysis related genes expression, including RBMX, YTHDF1, METTL3, IGF2BP2 (**F**) Kaplan-Meier curve of RBMX, YTHDF1, METTL3 *P < 0.05; *P < 0.05.

Modification of m6A plays a significant role in HCC. By analyzing TCGA and ICGC HCC data, we detected the correlation between the expression of ZNF320 and the expression of 21 m6A related genes in HCC, and ZNF320 expression significantly positively correlated with ZNF320, RBMX, RBM15B, LRPPRC, YTHDF1, HNRNPC (Figure 11A, P<0.01) in the TCGA database. Furthermore, ZNF320 expression significantly positively correlated with RBM15B, LRPPRC, YTHDF1, HNRNPC in ICGC data sets (Figure 11B, P < 0.01).

We divided TCGA samples into two groups according to the expression of ZNF320. We tried to exam the differential expression of genes related to M6A between the high and low ZNF320 groups. As shown in Figure 11, the m6A modification was not the same between high and low groups with the ZNF320 expression in HCC (Figure 11C). The intersection of genes with correlation coefficient greater than 0.39 with ZNF320 two databases was calculated, genes were presented in Venn’s diagram, including RBMX, YTHDF1, METTL3, IGF2BP2. (Figure 11D). The scattering plot shows the correlation between the expression of the genes related to ZNF320 and m6A (Figure 11E) Compared to the group of low expression, the expression of 19 genes in the high expression group of ZNF320 were increased (P <0.001). Then, we used Kaplan-Meier curve to reveal that the high expression of RBMX, YTHDF1, METTL3 were intensely associated with a poor prognosis of HCC (P<0.001) (Figure 11F). These results claim that ZNF320 may be closely related to the m6A modification of HCC, especially through its regulation with RBMX, YTHDF1, METTL3, which eventually influent the progression and prognosis of HCC.

## DISCUSSION

Liver cancer is the fifth most common type of cancer worldwide, it is also the second leading cause of death from cancer [23]. Due to the symptoms are not evident during early stages, A huge number of HCC patients are found in the advanced stages. Although many treatment strategies have been used [24], the prognosis of HCC patients are still not satisfactory. Furthermore, advanced patients are not amenable for surgery. Therefore, there exists an imperative need for effective early diagnostic markers which may be able to assist the existing clinical diagnoses to enhance the HCC patients’ prognosis. In our study, we figured out ZNF320 as a new potential prognostic biomarker for hepatocellular carcinoma., and studied the relationship between ZNF320 and cell cycle, tumor-associated immune cells, m6a.

With the purpose of detecting the expression and prognosis of ZNF320 in HCC, first through public database analysis, we noticed that ZNF320 expression in HCC tissues was highly increased compare with adjacent normal tissues, and we applied clinical samples to verify it. Secondly, we applied Kaplan-Meier survival analysis established that ZNF320 is one of the factors of poor overall survival and prognosis in HCC patients. At the same time, results showed that ZNF320 has been associated with many clinicopathological parameters, including tumor grade, tumor stage, and so on. Finally, univariate Cox analysis showed that ZNF320 was pointedly related to overall survival (OS). Variational calculation Cox analysis revealed that ZNF320 could be regarded as an independent predictor. At the same time, we explore the connection between ZNF320 methylation and ZNF320 expression through the cBioPortal dataset [25]. Results displayed that ZNF320 expression was positively associated with methylation (R = 0.1, p =0.022) in HCC, the methylation high levels of ZNF320 promoter in HCC were higher than that in normal tissue. Overall, these results manifested that ZNF320 is highly expressed in HCC, and the high expression of ZNF320 leads to a poor prognosis of HCC, the methylation with high level of ZNF320 promoter also result in a poor prognosis of HCC [26].

With the purpose of further exploring the function and mechanism of ZNF320 in HCC, we explored the co-expressed genes of ZNF320 in HCC through LinkedOmics, used GO and KEGG to perform functional analysis of the co-expressed genes, and performed GSEA analysis on ZNF320. The results indicated that the functional classification and KEGG pathway related to ZNF320 included: “FC epsilon pathway”, “FC gamma R mediated phagocytosis”, “cell cycle”, “WNT signaling pathway”, “pathway in cancer”, “leukocyte transendothelial migration”, “mismatch repair” and so on. In the results of GSEA, we noticed that ZNF320 can play a role by participating in DNA mismatch repair, so we deeply explored the correlation between ZNF320 and mismatch repair proteins, and the results showed that the expression of ZNF320 was associated with MSH2, MSH6, MLH1 and PMS2. All were positively correlated (Supplementary Figure 1A–1D), and we found that when MSH2 and MLH1 were highly expressed, HCC patients had a shorter survival time (Supplementary Figure 1E, 1F). It has also been reported before that mismatch repair-related genes are positively correlated with oncogene expression in HCC, and the role of oncogenes can affect the prognosis of patients. For example, MSH2 is positively correlated with oncogene expression [27], mismatch repair Repair-related gene EXO1 plays an oncogenic role in HCC [28], MSH6 is an up-regulated HCC staging-related gene, and high expression of MSH6 is positively correlated with 1-year recurrence of HCC [29], which is consistent with our results. In conclusion, the poor prognosis of HCC patients caused by the high expression of ZNF320 may be related to the high expression of MSH2, but whether ZNF320 leads to the poor prognosis of HCC through MLH1 and MSH2 needs more experiments to prove. However, more experiments are needed to prove whether ZNF320 leads to poor prognosis of HCC through MLH1 and MSH2. The main conclusion drawn from our research was that ZNF320 was closely related to cell cycle and immune infiltration.

The cell cycle is a highly regulated process that enables cell growth, replication of genetic material, and cell division [30]. In cancer, the genetic control of cell division has undergone a fundamental change, leading to unrestricted cell proliferation. The deregulation of cell cycle was related to cancer occur through abnormal expression of protein especially at different levels of the cell cycle [31, 32]. Studies have reported that KIF18A promotes the proliferation, invasion, and migration of liver cancer cells by promoting cell cycle signaling pathways [33]. At the same time, recent studies have found that KIF23 has carcinogenic effects in HCC [34, 35]. CKAP5 encodes a cytoskeleton-related protein belonging to the TOG/XMAP215 family. CKAP is also known as colon and liver tumor overexpression gene protein [34]. We used PPI to identify the first 13 genes with the highest cluster scores related to ZNF320 gene, and it turned out that six genes including SPDL1, KIF18A, PRIM2, GINS4, KIF23, CKAP5. What’s more, all of these genes play an important role in cell cycle. In addition, we used GEPIA to analyze the connection between expression of ZNF320 and SPDL1, KIF18A, PRIM2, GINS4, KIF23, CKAP5 and conducted the Kaplan–Meier survival method to dissect their prognosis. The results showed that SPDL1, KIF18A, PRIM2, GINS4, KIF23, CKAP5 were related to the ZNF320 expression and the high expression of SPDL1, KIF18A, PRIM2, GINS4, KIF23, CKAP5 lead to a low overall survival rate. This indicated that ZNF320’s influence on the prognosis of HCC may be related to the cell cycle.

What’s more, the disorder of the cell cycle is also related to the invasion, and migration of tumors. Through EdU and transwell assays, we found that interfering with the expression of ZNF320 could obviously inhibit the proliferation, invasion, and migration of HCC cells and downregulate the protein expression of CDK1, CDC20 and CCNB1. In summary, this experiment showed that down-regulation of ZNF320 can inhibit cell proliferation, invasion, and migration of HCC cells, which may be related to regulating the cell cycle signaling pathway.

With the advent of immunotherapy [36, 37], a shift in the way of treating cancer was created [38, 39]. Tumor microenvironment (TME) is one of the key factors affecting the efficacy of immunotherapy [40, 41]. In recent years, research have indicated that immune therapy provides a survival benefit for liver cancer treatment [42, 43]. Therefore, researching the immune microenvironment and identifying potential immunotherapeutic targets become increasingly important in improving the effectiveness of immunotherapy in patients. To some extent, this study explored the relationship between the expression levels of ZNF320 and immune infiltration levels, various immune markers. Positive relationships were found between the expression levels of ZNF320 and infiltration levels of some immune cells. Consistently, we found that ZNF320 is significantly related to the gene marker set of B cells, T cells (general), CD8+ T cells, TAM, M1 macrophages, and M2 macrophage immune cells. In general, our study demonstrated that ZNF320 may take a part in the regulation of infiltration cells in the immune microenvironment in HCC. Studies have shown that CD8+ T cells can kill tumor cells TAM to help tumor cells participate in immune escape. Although ZNF320 and CD8+ T cell infiltration are associated, we found that ZNF320 is positively correlated with the immune checkpoint gene (Supplementary Figure 2), cannot produce tumor killing benefits, and is actually involved in immune escape. It was found through TISIDB that ZNF320 is positively correlated with CCL26 while negatively correlated with CCL15 and CXCL2. Moreover, interference with ZNF320 can significantly affect the expression of these chemokines. To further explore the contribution of ZNF320 to immunotherapy, we further investigated the association of ZNF320 with immune checkpoint-related genes, and the results showed that ZNF320 was significantly positively correlated with PD-L1, meaning that patients with high expression of ZNF320 may benefit from PD-L1 blocking therapy. From the above, it demonstrates that ZNF320 acts a pivotal part in regulating the immune cell infiltration in HCC.

N6-methyladenosine, also called m6A, refers to the methylation of the sixth N position of adenylate in RNA, which mainly regulates RNA transcription, splicing, maturation, stability, translation, etc. The effect of m6A modification on human cancer has been confirmed in different cancer types [44], and the function of m6a modification on HCC has also received a lot of attention [45, 46]. Recent research have displayed that m6A modification have a significant role in HCC tumor, and many m6A-related genes can promote liver cancer progression [47, 48]. For example, YTHDF1 can promote the migration and invasion of HCC cells, and promote the proliferation of HCC cells by inducing EMT [49]. METTL3 is frequently upregulated in human HCC [50]. There is no research report on the communication between ZNF320 and m6A modification. Our results show that there is a strong correlation between ZNF320 and m6A. The 19 m6A-related genes showed differential expression when ZNF320 was overexpressed and under expressed. In addition, by constructing a Kaplan-Meier curve, it was found that compared to the low expression group, the survival time of the RBMX, YTHDF1, METTL3 high expression group was shorter. Our results show that the cancer-promoting impact of ZNF320 is associated with the modification of m6a, which may influent the mRNA methylation level of HCC through its association with YTHDF1, and finally impact the progress of HCC.

In conclusion, our research suggests that ZNF320 may be a possible biomarker for poor prognosis of hepatocellular carcinoma. ZNF320 may not only take part in cell cycle regulation and affect the proliferation of HCC, but also may assume the role in the microenvironment of HCC by regulating tumor infiltrating immune cells. In addition, the results claim that ZNF320 may be closely associated with the m6A modification of HCC, which eventually influents the progression and prognosis of HCC. These put forward that ZNF320 may serve as a target for early clinical diagnosis and treatment which may have a chance to improve diagnostic and therapeutic options, and at the same time provides a reference for further exploration of new cancer immunotherapy.

## Supplementary Material

Supplementary Figures

Supplementary Table 1
