# Supplementary material for: ZNF320 is a hypomethylated prognostic biomarker involved in immune infiltration of hepatocellular carcinoma and associated with cell cycle
Source: Aging (Albany NY). 2022 Oct 26;14(20):8411–36. doi: 10.18632/aging.204350 (PMC9648795; doi:10.18632/aging.204350)
Supplement: Supplementary Figures [file aging-14-204350-s001.pdf]

SUPPLEMENTARY FIGURES

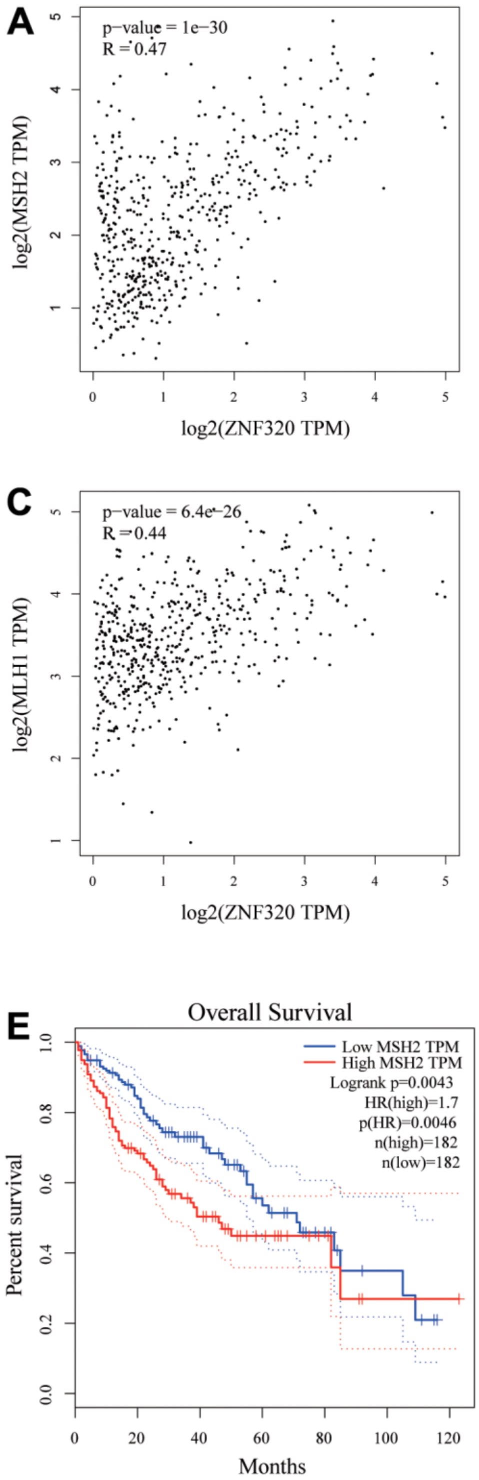

Supplementary Figure 1. Correlation between ZNF320 expression and mismatch repair proteins. (**A**) MSH2, (**B**) PMS2, (**C**) MLH1, (**D**) MSH6. (**E**, **F**). HCC patients with lower expression level of MSH2 and MLH1 had favorable OS results.

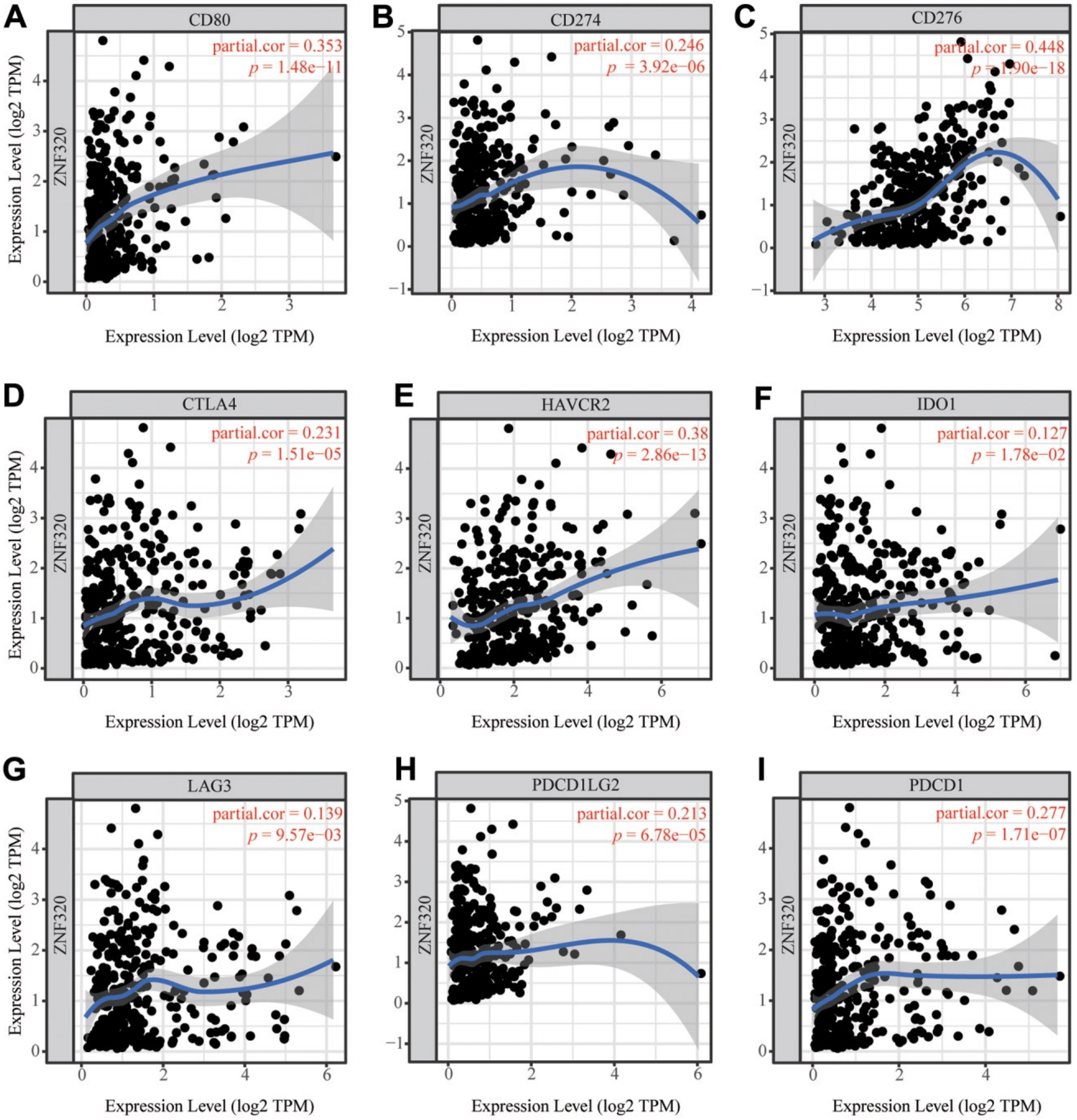

Supplementary Figure 2. ZNF320 expression correlates with the immune checkpoint gene in HCC. The association between the expression levels of ZNF320 and CD80 (**A**), CD274 (**B**), CD276 (**C**), CTLA4 (**D**), HAVCR2 (**E**), IDO1 (**F**), LAG3 (**G**), PDCDILG2 (**H**), and PDCD1 (**I**).
