# Supplementary material for: ZNF320 is a hypomethylated prognostic biomarker involved in immune infiltration of hepatocellular carcinoma and associated with cell cycle
Source: Aging (Albany NY). 2022 Oct 26;14(20):8411–36. doi: 10.18632/aging.204350 (PMC9648795; doi:10.18632/aging.204350)
Supplement: Supplementary Table 1 [file aging-14-204350-s002.docx]

**Supplementary Table 1. The top 200 genes that were correlated most clearly with ZNF320.**

| **Query** | **Statistic** |
| --- | --- |
| ZNF320 | 1 |
| ZNF816A | 0.867297442 |
| ZNF321 | 0.855147813 |
| ZNF468 | 0.789981575 |
| ZNF611 | 0.786112648 |
| ZNF28 | 0.77529904 |
| ZNF83 | 0.772791221 |
| ZNF525 | 0.765279372 |
| ZNF600 | 0.727046382 |
| ZNF14 | 0.722140665 |
| ZNF701 | 0.718192603 |
| ZNF808 | 0.704183627 |
| ZNF347 | 0.688330268 |
| ZNF160 | 0.685869857 |
| ZNF737 | 0.671466407 |
| ZNF665 | 0.653230266 |
| ZNF43 | 0.644109791 |
| ZNF135 | 0.644091907 |
| ZNF137 | 0.642102084 |
| ZNF528 | 0.640616167 |
| ZNF607 | 0.636841995 |
| ZSCAN18 | 0.633914084 |
| ZNF93 | 0.632209372 |
| ZNF85 | 0.62880708 |
| ZNF549 | 0.628193157 |
| ZNF702P | 0.626475156 |
| ZNF793 | 0.614238913 |
| ZFP82 | 0.613354284 |
| ZNF610 | 0.612695206 |
| ZNF506 | 0.611584647 |
| ZNF880 | 0.608692306 |
| ZNF826 | 0.597044291 |
| ZNF578 | 0.596363519 |
| ZNF845 | 0.58858374 |
| ZNF626 | 0.588482055 |
| ZNF681 | 0.587408954 |
| ZNF813 | 0.584419445 |
| ZNF879 | 0.581987485 |
| ZNF431 | 0.580043083 |
| ZNF256 | 0.57502037 |
| ZNF714 | 0.568434371 |
| ZNF239 | 0.567762664 |
| ZIK1 | 0.565278475 |
| ZNF695 | 0.559967554 |
| LOC441666 | 0.559707063 |
| ZNF738 | 0.557522013 |
| MTMR2 | 0.553703771 |
| ZNF471 | 0.55206442 |
| ZNF486 | 0.541913682 |
| ZNF391 | 0.537219253 |
| ZNF667 | 0.536984316 |
| ZNF418 | 0.536274582 |
| ZNF430 | 0.535409655 |
| ZNF761 | 0.531556989 |
| FAM64A | 0.529967319 |
| ZNF300 | 0.529155583 |
| ZNF765 | 0.527404971 |
| ZNF415 | 0.524508333 |
| ZFP28 | 0.524232712 |
| MAPRE1 | 0.52255028 |
| LOC147804 | 0.513727217 |
| ZNF354A | 0.513530054 |
| ZNF568 | 0.509371748 |
| ZNF257 | 0.508392916 |
| ZNF829 | 0.504811312 |
| C17orf63 | 0.500268191 |
| ZNF781 | 0.49970728 |
| PNMA1 | 0.495794813 |
| ZNF426 | 0.493715756 |
| ZNF439 | 0.492217244 |
| ZDHHC13 | 0.491730065 |
| ZNF287 | 0.491655246 |
| TRDMT1 | 0.488827027 |
| ZNF98 | 0.488079616 |
| ZNF675 | 0.4878047 |
| ZNF141 | 0.487670282 |
| FRAS1 | 0.486653561 |
| ZNF285 | 0.485466827 |
| ZNF788 | 0.484604536 |
| ZNF559 | 0.484281152 |
| C18orf54 | 0.483792827 |
| ZNF331 | 0.483549666 |
| COMMD2 | 0.482346529 |
| ZNF708 | 0.479069091 |
| HIST3H2A | 0.478600148 |
| ZNF501 | 0.476749109 |
| ZNF124 | 0.47577913 |
| PCLO | 0.472956256 |
| PRKCI | 0.471376423 |
| ZNF71 | 0.471118009 |
| ZNF273 | 0.470129528 |
| ENPP5 | 0.467149175 |
| ZNF100 | 0.466898703 |
| ZNF682 | 0.466871588 |
| ZNF354C | 0.466541366 |
| ZNF204P | 0.465915451 |
| ZNF773 | 0.465150523 |
| WNK2 | 0.463948014 |
| ABCC10 | 0.463442175 |
| ZNF107 | 0.462758459 |
| ZNF492 | 0.462069946 |
| YEATS2 | 0.46080442 |
| ZNF727 | 0.46077594 |
| PRSS16 | 0.460467292 |
| TRIM59 | 0.460004644 |
| RCC2 | 0.458606672 |
| ZNF329 | 0.458419171 |
| ZNF253 | 0.457523425 |
| TMED3 | 0.456368008 |
| ZNF614 | 0.45538832 |
| TET1 | 0.45479798 |
| ZNF606 | 0.454773578 |
| FAM164A | 0.454033716 |
| MAPK13 | 0.45372117 |
| CSNK2A1 | 0.453351311 |
| ACTL6A | 0.453052982 |
| PLEKHB1 | 0.451788667 |
| ZNF550 | 0.451572499 |
| NCK2 | 0.451287955 |
| ZNF519 | 0.451141805 |
| ZBTB12 | 0.450192568 |
| MPP2 | 0.449693608 |
| ZNF585A | 0.448709227 |
| ZFP30 | 0.447997224 |
| JRKL | 0.445482812 |
| ZNF70 | 0.444268967 |
| C2orf29 | 0.443666134 |
| NCBP2 | 0.442707044 |
| VPS37C | 0.442605173 |
| ZNF772 | 0.442595786 |
| DVL3 | 0.442275981 |
| HCG18 | 0.441577269 |
| PLAGL2 | 0.441175111 |
| ZNF676 | 0.441120989 |
| ZNF470 | 0.440877343 |
| ZNF649 | 0.439562416 |
| GTF3C2 | 0.439302613 |
| TRAM1L1 | 0.43930241 |
| C9orf140 | 0.438984088 |
| CLSTN1 | 0.436042654 |
| ROD1 | 0.435718644 |
| ZNF711 | 0.435165952 |
| ILDR1 | 0.435051414 |
| BTBD3 | 0.434755834 |
| SOX4 | 0.434284992 |
| ACOT11 | 0.43408956 |
| SLC25A24 | 0.433862022 |
| CNOT6 | 0.432328884 |
| ZNF233 | 0.432272056 |
| CCDC99 | 0.431607198 |
| ECT2 | 0.430935073 |
| STX6 | 0.428938242 |
| DLX4 | 0.428017127 |
| RCAN3 | 0.427598882 |
| KIF3C | 0.427334361 |
| RTKN2 | 0.427162258 |
| CDK16 | 0.427142536 |
| PKM2 | 0.427117249 |
| ZNF835 | 0.426539544 |
| FBLIM1 | 0.42651762 |
| ZNF229 | 0.426292811 |
| C1orf116 | 0.425962723 |
| ZNF283 | 0.425805914 |
| EVC2 | 0.425110734 |
| PRAME | 0.425027813 |
| ZNF211 | 0.424405381 |
| ZNF502 | 0.424295922 |
| VPS24 | 0.423859387 |
| C3orf52 | 0.423834857 |
| PDCL | 0.4229629 |
| PLEKHG4 | 0.422660854 |
| ZNF530 | 0.422215794 |
| NFYA | 0.422177229 |
| STX3 | 0.422066855 |
| IFT52 | 0.421848703 |
| HKR1 | 0.421339304 |
| PRKCD | 0.421253338 |
| PPT1 | 0.420220976 |
| KIF12 | 0.419810788 |
| SPIN1 | 0.41886887 |
| DNMT3B | 0.41814737 |
| C1orf88 | 0.417958887 |
| ZNF518B | 0.417095439 |
| NAP1L3 | 0.416057865 |
| SH3PXD2B | 0.415625389 |
| PRIM2 | 0.415613497 |
| RALGAPB | 0.415551037 |
| MARK2 | 0.415443809 |
| CKAP5 | 0.414291383 |
| C3orf21 | 0.41393503 |
| C5orf13 | 0.413549517 |
| ZNF208 | 0.413476817 |
| ZNF493 | 0.413237606 |
| EPCAM | 0.412835047 |
| ZNF585B | 0.412540394 |
| ASNS | 0.411895537 |
| TMEM87B | 0.411497075 |
| PPAP2C | 0.411351954 |
| BCORL1 | 0.410976629 |
| RYK | 0.410752137 |
| SERPINH1 | 0.410032479 |
